# 10-Hydroxydec-2-Enoic Acid Reduces Hydroxyl Free Radical-Induced Damage to Vascular Smooth Muscle Cells by Rescuing Protein and Energy Metabolism

**DOI:** 10.3389/fnut.2022.873892

**Published:** 2022-05-26

**Authors:** Pei Fan, Fangfang Sha, Chuan Ma, Qiaohong Wei, Yaqi Zhou, Jing Shi, Jiaojiao Fu, Lu Zhang, Bin Han, Jianke Li

**Affiliations:** ^1^Institute of Apicultural Research/Key Laboratory of Pollinating Insect Biology, Ministry of Agriculture and Rural Affairs, Chinese Academy of Agricultural Sciences, Beijing, China; ^2^College of Biological Engineering, Henan University of Technology, Zhengzhou, China

**Keywords:** royal jelly, 10-hydroxydec-2-enoic acid, hydroxyl radical, vascular smooth muscle cell, proteomics

## Abstract

10-Hydroxydec-2-enoic acid (10-HDA), an unsaturated hydroxyl fatty acid from the natural food royal jelly, can protect against cell and tissue damage, yet the underlying mechanisms are still unexplored. We hypothesized that the neutralization of the hydroxyl free radical (•OH), the most reactive oxygen species, is an important factor underlying the cytoprotective effect of 10-HDA. In this study, we found that the •OH scavenging rate by 10-HDA (2%, g/ml) was more than 20%, which was achieved through multiple-step oxidization of the –OH group and C=C bond of 10-HDA. Moreover, 10-HDA significantly enhanced the viability of vascular smooth muscle cells (VSMCs) damaged by •OH (*P* < 0.01), significantly attenuated •OH-derived malondialdehyde production that represents cellular lipid peroxidation (*P* < 0.05), and significantly increased the glutathione levels in •OH-stressed VSMCs (*P* < 0.05), indicating the role of 10-HDA in reducing •OH-induced cytotoxicity. Further proteomic analyses of VSMCs identified 195 proteins with decreased expression by •OH challenge that were upregulated by 10-HDA rescue and were primarily involved in protein synthesis (such as translation, protein transport, ribosome, and RNA binding) and energy metabolism (such as fatty acid degradation and glycolysis/gluconeogenesis). Taken together, these findings indicate that 10-HDA can effectively promote cell survival by antagonizing •OH-induced injury in VSMCs. To the best of our knowledge, our results provide the first concrete evidence that 10-HDA-scavenged •OH could be a potential pharmacological application for maintaining vascular health.

## Introduction

Royal jelly (RJ), a secretion from the hypopharyngeal and mandibular glands of worker honeybees, has been widely regarded as a functional food for the promotion of human health (1). Among the components of RJ, 10-hydroxydec-2-enoic acid (10-HDA) is a unique substance exclusively found in RJ at a concentration of 1.4–2.0% (2). Accumulating evidence suggests that 10-HDA has a wide range of bioactivities, including antibacterial, anti-inflammatory, and antitumor effects (3–6). Furthermore, 10-HDA has been shown to prevent cell and tissue photoaging damage induced by ultraviolet irradiation by enhancing collagen production in human dermal fibroblasts (7). 10-HDA also alleviated lipopolysaccharide (LPS)-induced blood–brain barrier damage in mice (8). Our previous work established that 10-HDA attenuated cyclophosphamide-induced weight loss in the mouse thymus and spleen and thus has the potential to fortify immunity (9). Although the emerging roles of 10-HDA against cell damage have been demonstrated in different cell types, the molecular mechanisms still remain largely unknown.

Oxygen free radicals, such as the hydroxyl radical (•OH) and superoxide anion radical (${\text{O}}_{2}{•}^{-}$), are major reactive oxygen species (ROS). ROS are involved in a variety of cell processes and diseases and are highly reactive (10). ROS can also damage DNA, proteins, and lipids. In particular, the cell membrane is susceptible to ROS attack and lipid peroxidation, which impairs cellular structures and functions (11). The alkylating agent and chemotherapeutic cyclophosphamide can be metabolized into acrolein, a substance that can produce a large quantity of ROS (12). Therefore, our previous data in which 10-HDA was found to mitigate immune organ damage caused by cyclophosphamide may potentially result from the role of 10-HDA in removing ROS (9). Among all the oxygen free radicals, •OH is the most potent and non-selective, and it is generated in a number of conditions *in vivo* in which it can trigger severe cell damage (13–15). Hence, we hypothesized that •OH scavenging by 10-HDA could be the reason for the reduction of cell damage caused by cyclophosphamide.

Cardiovascular disease is one of the greatest risks to human health worldwide. Vascular smooth muscle cells (VSMCs) are the major cells in the blood vessel wall, controlling vascular movement *via* contraction and relaxation. Dysfunction of VSMCs leads to delayed development of blood vessels and thus may cause embryonic and post-natal abnormalities (16), reflecting the fact that VSMC survival is directly linked to vascular health. Oxidative stress of VSMCs resulting from high ROS levels has been associated with cardiovascular diseases, including atherosclerosis, hypertension, and stenosis (17). However, different ROS exert distinct effects on VSMCs, in which •OH, but not ${\text{O}}_{2}{•}^{-}$, causes the death of VSMCs (18, 19), making VSMCs an appropriate model for investigating specific ROS-induced cell damage. Several substances, such as gallic acid, induced VSMC death *via* the generation of •OH (19). Therefore, in this study, the capability of 10-HDA to scavenge •OH was tested first using the Fenton system, commonly used to investigate ROS-induced cell impairment *in vitro*, in which •OH is produced by Fe^2+^ and H_2_O_2_ (15). Then, a model of •OH-damaged VSMCs was established to uncover the underlying mechanisms of 10-HDA in survival protection, casting light on the potential of 10-HDA as a new agent for retaining vascular health.

## Materials and Methods

### Major Chemicals and Materials

The major chemicals and materials are specified in the following sections.

### Measurement of the Scavenging Rate of •OH by 10-HDA

To determine the role of 10-HDA in •OH removal, the Hydroxyl Free Radical Scavenging Capacity Assay Kit (Catalog No. R30345, Yuanye Bio Sci & Tech Co., Ltd, Shanghai, China), based on the Fenton/1,10-phenanthroline monohydrate colorimetric method, and the Hydroxyl Free Radical Assay Kit (Catalog No. A018-1-1, Nanjing Jiancheng Bioengineering Institute, Nanjing, China), based on the Fenton/Griess reagent method, were used to measure the scavenging rate of •OH. In brief, 0.02 g 10-HDA (Yuanye Bio Sci & Tech Co., Ltd., Shanghai, China) was dissolved in 200 μl ethanol, followed by the addition of 800 μl distilled water, to a final concentration of 2% (g/ml) 10-HDA, which approximates its concentration in RJ. Then, 400 μl of the 10-HDA solution was added to the kit reaction system, and the solution absorbance, which was later used to calculate the scavenging rate of •OH, was assayed at the wavelength of 536 nm *via* a visible spectrophotometer (Shanghai Metash Instrument Co., Ltd., Shanghai, China). A 400 μl sample of solution without adding 10-HDA was prepared as the control sample. A sample containing 0.02 g (1,000 IU/g) vitamin E (Beijing Solarbio Science & Technology Co., Ltd., Beijing, China) was used as a positive control.

### Product Identification From •OH and 10-HDA Reactions

To gain more evidence of 10-HDA removal of •OH, high-performance liquid chromatography (HPLC) was used to trace concentration changes after adding •OH to the 10-HDA solution, which is a direct observation of 10-HDA reacting with •OH. The reaction system (i.e., 10-HDA + •OH sample) (10 ml), containing 0.2 g 10-HDA, 2 ml ethanol, 0.033 g FeSO_4_•7H_2_O, and 3.3 ml H_2_O_2_, was incubated at 37°C for 3 h. For the 10-HDA control sample (10-HDA sample) (10 ml), H_2_O_2_ was substituted with the same volume of H_2_O. Both samples were diluted with ethanol at a 1:1 ratio prior to the analysis. The HPLC analysis was performed using a Waters e2695 platform (Waters Corp., Milford, MA, USA) with a C18 column (5 μm, 4.6 mm × 250 mm) (TupLabs, Tianjin, China), in which the mobile phase was methanol and 0.1% formic acid (55:45, v/v), the flow rate was 0.5 ml/min at room temperature, and the detection wavelength was 210 nm for 10-HDA, as reported in a previous study (20).

### Qualitative Analysis of the Products of 10-HDA Reacted With •OH

To characterize the products from the reaction between 10-HDA and •OH, the 10-HDA sample and the 10-HDA + •OH sample (3 μl each), both of which were prepared as described in the “Product identification from •OH and 10-HDA reactions” section, were assayed *via* ultra-HPLC-high-resolution mass spectrometry (UHPLC-HRMS) as previously described (21). In brief, the liquid chromatographic separation was performed on a Dionex UltiMate 3000 system (Thermo Fisher Scientific, Inc., Waltham, MA, USA) using reverse-phase liquid chromatography (RPLC) with a positive/negative polarity switching mode. The RPLC separation was conducted on a ZORBAX SB-Aq C18 column (100 mm × 2.1 mm, 1.8 μm; Agilent Technology) at 40°C with mobile phase A (i.e., 0.1% formic acid in water) and B (i.e., 0.1% formic acid in acetonitrile). Gradient elution with a flow rate of 0.3 ml/min was set as follows: 0–2 min, 95–70% A; 2–8 min, 70–15% A; 8–9 min, 15–15% A; 9–9.5 min, 15–95% A; and 9.5–13 min, 95–95% A. A Q-Exactive mass spectrometer (MS) (Thermo Fisher Scientific, Inc., Waltham, MA, USA) equipped with a heated electrospray ionization source was employed for MS analysis. The raw data were analyzed with the Qual Browser in the Xcalibur software (version 4.0, Thermo Fisher Scientific, Inc., Waltham, MA, USA), in which the mass tolerance was set to 5.0 ppm, the scan filter settings were FTMS-p ESI FULL ms, and the plot type was selected as base peak.

### Cell Culture

Mouse VSMCs (an immortalized C57BL/6 strain) were purchased from American Type Culture Collection. The cells were cultured in the Dulbecco's modified Eagle's medium (Gibco, Thermo Fisher Scientific, Inc., Waltham, MA, USA) with 10% fetal bovine serum at 37°C and 5% CO_2_ (Zhejiang Tianhang Biotechnology Co., Ltd, Huzhou, Zhejiang, China).

### VSMC Viability Comparison by Morphological Imaging and 3-(4,5-Dimethylthiazol-2-Yl)-2,5-Diphenyltetrazolium Bromide Assay

To investigate the role of 10-HDA in protecting against •OH-induced cytotoxicity, the VSMCs were subjected to a Fenton reaction system to generate •OH damage. For the Fenton reaction system, FeSO_4_•7H_2_O and H_2_O_2_ were added to the VSMC culture media to final concentrations of 0.1 mM and 1 mM, respectively, to expose the cells to •OH. To analyze the protective effects of 10-HDA on VSMCs, the cells were treated with 10-HDA prior to the •OH exposure, based on previously reported methods with some modifications (5, 22). In brief, VSMC media were supplemented with 10-HDA dissolved in ethanol (i.e., 3 mM) for 30 min prior to the addition of the Fenton system. Importantly, to account for the potential adverse effects of ethanol on cells and 10-HDA's ability to reduce media pH, the control and the •OH-damaged VSMCs were also treated with the same volume of ethanol, and the pH of the media was adjusted to that of the 10-HDA-supplemented VSMCs using HCl. Thereafter, all three groups of VSMCs (i.e., control, •OH-damaged, and 10-HDA-supplemented) were incubated at 37°C for 3 h.

To determine whether 10-HDA attenuates •OH-induced VMSC damage, the morphologies of the control, •OH-damaged, and 10-HDA-supplemented VSMCs were imaged and compared. First, VSMCs (5 × 10^5^ cells/well) were seeded in a 6-well plate and cultured for 24 h. After treatment of •OH or •OH + 10-HDA at 37°C for 3 h in 2 ml media, the three groups of VSMCs were photographed under an inverted microscope using a 10 × 40 magnification.

To further investigate 10-HDA's ability to reduce VMSC damage by •OH, the 3-(4,5-dimethylthiazol-2-yl)-2,5-diphenyltetrazolium bromide (MTT) assay (Beijing Solarbio Science & Technology Co., Ltd., Beijing, China) was used to compare the viability of the VSMCs. VSMCs were seeded in a 96-well plate (5 × 103 cells/well). After 24 h, the media were replaced by 100 μl fresh media that contained treatment of •OH or •OH + 10-HDA. After incubation at 37°C for 3 h, 20 μl MTT (5 mg/ml) was added to each well, and the cells were incubated for another 4 h. Finally, the cells were gently shaken with 100 μl dimethyl sulfoxide for 15 min in the dark. The absorbance of each well was assayed at 490 nm *via* a microplate reader (Tecan Group Ltd., Männedorf, Switzerland).

### Cell Lipid Peroxidation Assay Through Malondialdehyde Determination

To elucidate the mechanism of how 10-HDA restores the viability of •OH-damaged VSMCs, the levels of cell lipid peroxidation, usually reflected by MDA production, were measured. The VSMCs were seeded in a 24-well plate at 1 × 10^5^ cells/well and cultured for 24 h. The cells were then treated with •OH or •OH + 10-HDA at 37°C for 3 h. Furthermore, 10 μl of the cell supernatant was used to determine the MDA level using an MDA assay kit (Catalog No. R21870, Yuanye Bio Sci & Tech Co., Ltd., Shanghai, China). After the reaction, the absorbance was assayed at 535 nm to calculate MDA concentration, according to the kit manufacturer's instructions. The MDA level in the lysate of the VSMCs was also determined by using a 6-well plate with 3 × 10^5^ cells/well, with the same •OH or •OH + 10-HDA treatment, and the same assay kit.

### Glutathione (Reduced) Assay in VSMCs

To evaluate the effects of 10-HDA against oxidative stress from •OH, the levels of reduced glutathione, an endogenous antioxidant in VSMCs, were determined. VSMCs were seeded at 3 × 10^5^ cells/well in a 6-well plate and then treated with •OH or •OH + 10-HDA, using the same treatment as described in the “VSMC viability comparison by morphological imaging and 3-(4,5-dimethylthiazol-2-yl)-2,5-diphenyltetrazolium bromide assay” section. The reduced glutathione contents in VSMCs were measured using the Reduced Glutathione Assay Kit (Catalog No. BC1175, Beijing Solarbio Science & Technology Co., Ltd., Beijing, China).

### Proteomic Analysis of VSMCs With •OH Cytotoxicity and 10-HDA Rescue

To explore the mechanisms of 10-HDA protection against •OH toxicity, a liquid chromatography-tandem mass spectrometry (LC-MS/MS)-based proteomic strategy was applied. The control, •OH-damaged, and 10-HDA-rescued VSMCs were obtained according to the method described in the “VSMC viability comparison by morphological imaging and 3-(4,5-dimethylthiazol-2-yl)-2,5-diphenyltetrazolium bromide (MTT) assay” section. For each group, three replicates of cell samples were prepared.

To extract protein, the VSMCs were homogenized in lysis buffer at a cell:lysis buffer ratio of 1:10 (mg/μl) (23) and ultrasonicated on ice. After centrifugation at 12,000 rpm for 15 min, the supernatant was collected. Furthermore, three times the supernatant volume of ice-cold acetone was added, and the sample was incubated for 30 min to precipitate the proteins, which were then isolated by centrifugation at 8,000 rpm for 15 min. The proteins were dried for 10 min to remove the acetone, redissolved in 80 μl urea (5 M), and mixed with 320 μl NH_4_HCO_3_ (40 mM). The protein concentration was assayed *via* the Bradford method. Next, 200 μg protein samples were reduced using dithiothreitol (DTT) (100 mM) at the ratio of 10:1 (v/v) protein solution: DTT for 1 h at room temperature, followed by alkylation in iodoacetamide (100 mM) in the dark for 1 h using five times the volume of DTT. Samples were then digested at 37°C for 12 h to prepare peptides using 4 μg sequencing grade-modified trypsin (Promega Corporation, Madison, WI, USA). The trypsinization was terminated using 1 μl formic acid. The peptides were desalted using C18 columns (Thermo Fisher Scientific, Inc., Waltham, MA, USA) and dried *via* a rotary evaporator. Finally, the peptides were redissolved in 0.1% formic acid at a concentration of 0.25 μg/μl.

Digested samples (8 μl) were analyzed in a Q-Exactive HF MS (Thermo Fisher Scientific, Inc., Waltham, MA, USA), using the same parameters for the HPLC and MS as previously reported (24). The raw data retrieved from the Xcalibur software (version 4.0, Thermo Fisher Scientific, Inc., Waltham, MA, USA) were searched using the PEAKS software (version 8.5, Bioinformatics Solutions Inc., Waterloo, ON, Canada) against a database containing 86,618 protein sequences of *Mus musculus* from Uniprot (https://www.uniprot.org) released in January 2021 and the common Repository of Adventitious Proteins (cRAP, downloaded from The Global Proteome Machine Organization). Protein abundance was determined by label-free quantification (LFQ) between groups, in which the protein peptides screened for LFQ contained at least two unique peptides, with at least two samples per group. The protein fold change was set to ≥ 1.5 with a significance of ≥ 13. The significance method used was the analysis of variance (ANOVA). The differentially expressed proteins were identified in protein groups.

### Bioinformatical Analysis

To interpret the molecular basis of 10-HDA in relieving the •OH-damaged VSMC malfunction at the protein level, the proteins that were significantly downregulated by •OH and significantly upregulated with the 10-HDA treatment were used for the enrichment analysis with Gene Ontology (GO) for GO terms and the Kyoto Encyclopedia of Genes and Genomes (KEGG) for pathways through the online tool KOBAS-i (kobas.cbi.pku.edu.cn) (25). The Uniprot IDs for the proteins were analyzed using the hypergeometric test/Fisher's exact test as the statistical method, and the false discovery rate was corrected by the Benjamini and Hochberg procedure. The term or pathway with a corrected *P*-value <0.05 was considered significant.

To expound on the relationships between key proteins that might be regulated by 10-HDA in the •OH-damaged VSMCs, the protein-protein interaction (PPI) networks for these proteins were constructed using the online software NetworkAnalyst 3.0 (www.networkanalyst.ca) (updated 2021-05-18) (26). In brief, the generic PPI networks were generated based on the STRING Interactome with medium (400)-high (1,000) confidence scores (the score cutoff was set as 900). The subnetworks that had at least three nodes were shown.

To interpret the distribution of proteins downregulated by •OH and upregulated by 10-HDA in VSMCs, the online tool Cell-PLoc 2.0 (www.csbio.sjtu.edu.cn/bioinf/Cell-PLoc-2) was used to predict the protein subcellular localization (27). The amino acid sequences of the proteins were downloaded from the UniProt database and analyzed in Euk-mPLoc 2.0, a sub-module in Cell-PLoc 2.0, for the prediction of eukaryotic proteins.

### Intracellular Adenosine-Triphosphate Assay of VSMCs

To determine whether 10-HDA boosts energy production when exposed to •OH oxidative stress in VSMCs, the intracellular ATP levels of the control, •OH-damaged, and •OH + 10-HDA-supplemented VSMCs were assayed and compared. The VSMCs were similarly treated as in Section Glutathione (Reduced) Assay in VSMCs and then analyzed using an ATP assay kit (Catalog No. A095-1-1, Nanjing Jiancheng Bioengineering Institute), following the manufacturer's protocol.

### Western Blotting

To verify the relative expression levels of histone deacetylase 1 (HDAC1) and heterogeneous nuclear ribonucleoprotein D0 (HNRNPD) changed by •OH and 10-HDA, the Western blotting method was applied using reduced glyceraldehyde-phosphate dehydrogenase (GAPDH) and β-tublin as internal references. The VSMCs were sampled the same as that in the proteomic analysis, and they were homogenized in the RIPA buffer (Beijing Solarbio Science & Technology) with 1% phenylmethylsulfonyl fluoride (the protease inhibitor) on ice for 20 min. After centrifugation at 13,000 rpm for 10 min, the proteins contained in the supernatants were obtained and denatured at 95°C for 5 min with 5 × loading buffer. For detecting HDAC1, the concentrations of spacer gel and separation gel for electrophoresis were 5 and 12%, respectively. For HNRNPD, those were 4 and 9%, respectively. The proteins were then transferred to the nitrocellulose (NC) membrane under 110 mA for 180 min (i.e., HDAC1 and HNRNPD) or 150 min (i.e., GAPDH and β-tublin). The NC membrane was blocked in the phosphate-buffered solution with 0.05% Tween-20 (PBST) with 5% fat-free milk for 1 h, followed by the incubation of the primary antibody against HDAC1 (Proteintech Group, Inc., Rosemont, IL, USA), HNRNPD (Proteintech Group, Inc., Rosemont, IL, USA), GAPDH (Sigma-Aldrich Corp., St Louis, MO, USA), or β-tublin (Abbkine Scientific Co., Ltd., Wuhan, Hubei, China) in PBST overnight at 4°C and the IRDye^®^ 680RD Goat anti-rabbit or anti-mouse IgG secondary antibody (LI-COR, Inc., Lincoln, NE, USA) in PBST containing 5% fat-free milk for 1 h at room temperature. The bands were visualized using the Odyssey^®^ CLx Imaging System (LI-COR, Inc., Lincoln, NE, USA), and the band intensities were quantified using the ImageJ software.

### Statistical Analysis

Apart from the proteomic and bioinformatic statistics, the experimental data were represented by the mean ± standard deviation (SD) based on at least three independent trials. One-way ANOVA was used to test pairwise differences in the scavenging rate of •OH. The Student's *t*-test was used to determine the differences between the control and the •OH-damaged VSMCs, or the •OH-damaged and the •OH + 10-HDA-supplemented VSMCs, in which, *P* < 0.05 was significant.

## Results

### 10-HDA Scavenges •OH *via* a Series of Oxidation Reactions

**Figure 1:**
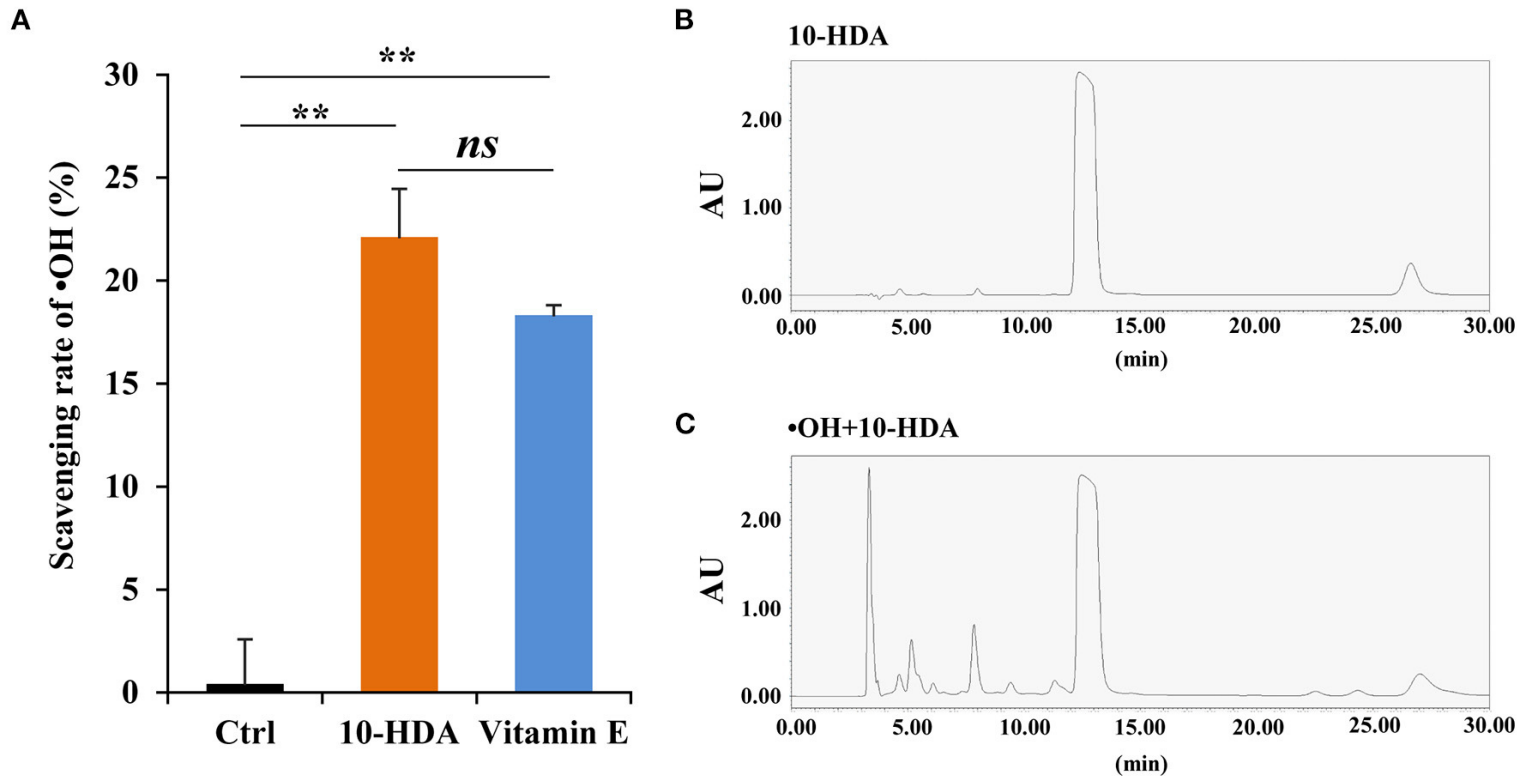
10-Hydroxydec-2-enoic acid (10-HDA) is capable of scavenging •OH. **(A)** The scavenging rates of •OH (generated from the Fenton system) by control, 10-HDA (2%), and vitamin E (2%, positive control) *via* the Fenton reaction-based colorimetric method. The scavenging rates were shown as mean ± standard deviation (SD) (*n* = 3; **P < 0.01; *ns*, no significance, represents *P* > 0.05). **(B)** The only peak for the 10-HDA sample without •OH addition in the high-performance liquid chromatography (HPLC) analysis at 210 nm. **(C)** The multiple peaks in the chromatogram at 210 nm, which indicates that more compounds emerged after 10-HDA (2%) were treated with •OH at 37°C for 3 h.

To test whether 10-HDA is capable of eliminating •OH, the scavenging rate of •OH was assayed using a 10-HDA solution (2%). The scavenging rate was more than 20%, significantly higher than that of the control (*P* < 0.01) based on the Fenton/1,10-phenanthroline monohydrate method, indicating that 10-HDA can remove •OH from the solution. Compared with the positive control (i.e., 2% vitamin E), the scavenging rate of •OH by 10-HDA was slightly higher, but the difference was statistically insignificant (*P* > 0.05) (Figure 1A). The second assay approach using the Fenton/Griess reagent also showed that 10-HDA had a significantly higher scavenging ability for •OH than the control (*P* < 0.001) (Supplementary Figure S1). In the HPLC analysis, a single peak (Figure 1B) generated by 10-HDA confirmed that the solution was free of •OH. However, the presence of a string of additional peaks (Figure 1C) indicated new products that may be due to the addition of •OH to 10-HDA. Taken together, 10-HDA could scavenge •OH, which may be the result of their direct reaction.

**Figure 2:**
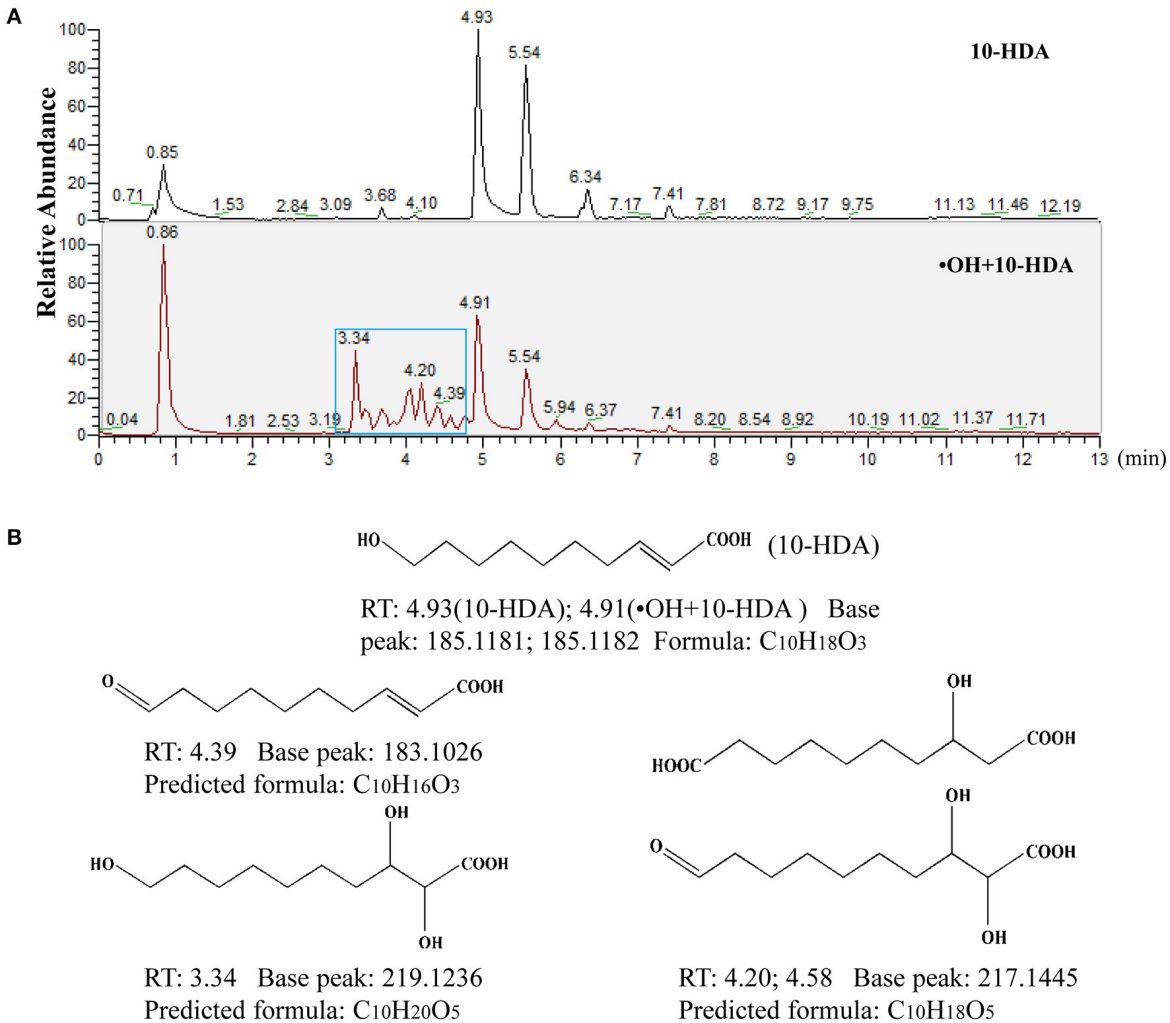
10-Hydroxydec-2-enoic acid (10-HDA) likely undergoes a set of oxidative reactions to eliminate •OH. **(A)** The comparison of ultra-high-performance liquid chromatography-high-resolution mass spectrometry (UHPLC-HRMS) chromatograms between 10-HDA and •OH + 10-HDA reaction product samples. The blue box indicates the peaks of new compounds after the reaction of •OH with 10-HDA. **(B)** The major predicted structures and formulas of the reaction products with molecular weights according to the MS base peaks implicated in the chromatographic peaks. Retention time (RT) represents the presence of the predicted reaction products in the 13 min elution gradient.

To further unravel the mechanism of the 10-HDA and •OH reaction, UHPLC-HRMS was used to identify the products after the addition of •OH to the 10-HDA solution. Both the 10-HDA and the •OH + 10-HDA samples had chromatographic peaks for 10-HDA (retention time (RT) at 4.93 and 4.91 min, respectively) (Figure 2A), in which the MS base peaks at m/z = 185.1181 and 185.1182 in the negative ion mode were implicated (Supplementary Figure S2A). Compared with the chromatogram of the 10-HDA sample without •OH, a string of peaks emerged after 10-HDA was treated with •OH, indicating potentially derived substances (Figure 2A). Within these extra chromatographic peaks, the MS base peaks at m/z = 183.1026 (RT at 4.39 min), 217.1445 (RT at 4.20 and 4.58 min), and 219.1236 (RT at 3.34 min) represented the major predicted oxidized products (Supplementary Figure S2B), with the possible molecular formulas C_10_H_16_O_3_ (the oxidation of the hydroxyl group (–OH) to a carbonyl group (C=O)), C_10_H_18_O_5_ (the oxidation of –OH to a carboxyl group (-COOH) and the hydroxylation of the double-double carbon bond (C=C) or the oxidation of –OH to C=O and the dihydroxylation of C=C)), and C_10_H_20_O_5_ (the dihydroxylation of C=C), respectively (Figure 2B). Therefore, 10-HDA may undergo a series of oxidation reactions to scavenge •OH.

### 10-HDA Protected VSMC Viability Against •OH Toxicity

**Figure 3:**
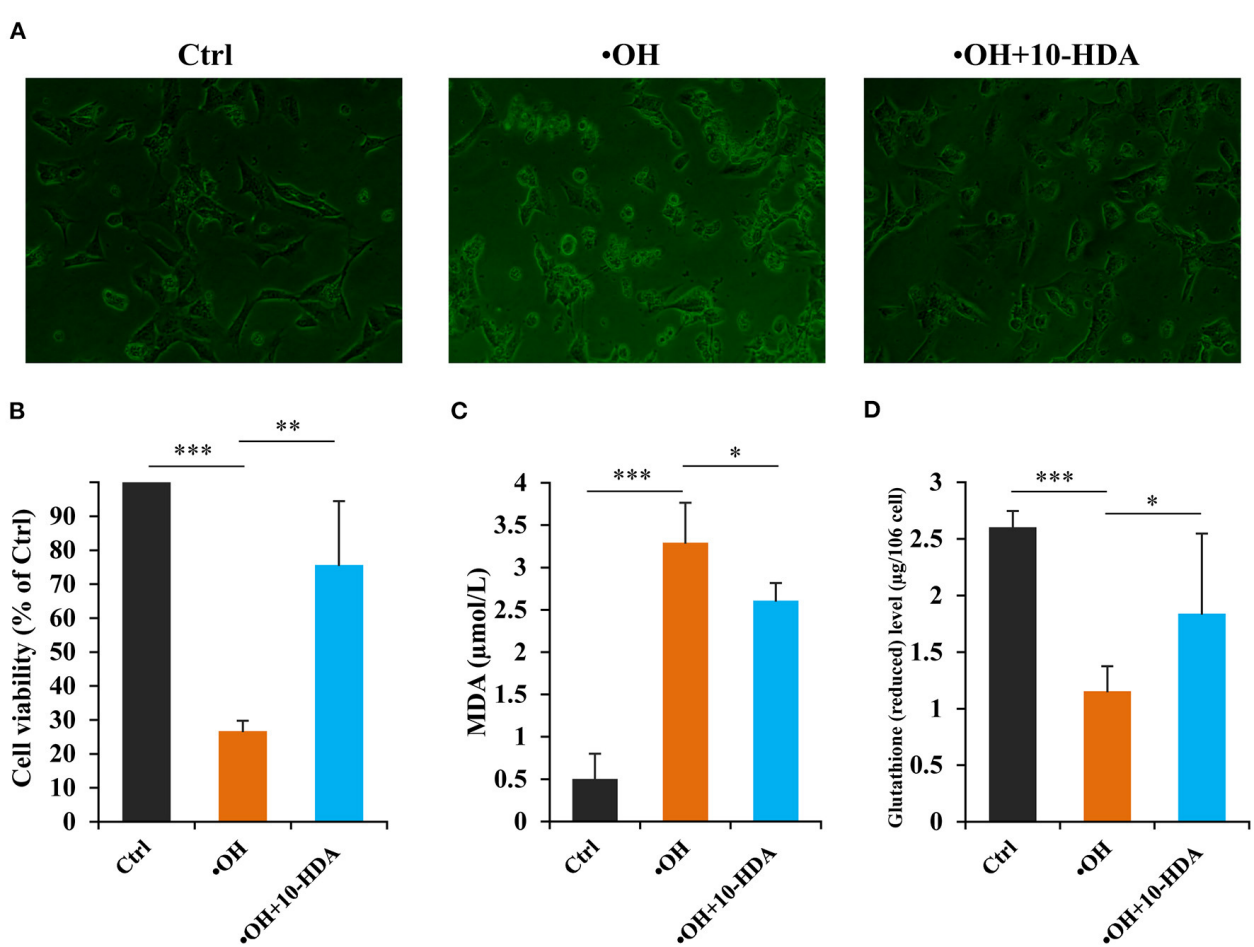
10-Hydroxydec-2-enoic acid (10-HDA) attenuates •OH-induced damage in vascular smooth muscle cells (VSMCs). **(A)** The morphological comparison between the control, •OH-damaged (0.1 mM FeSO_4_•7H_2_O + 1 mM H_2_O_2_), and 10-HDA-supplemented (3 mM) VSMCs. **(B)** The viabilities of •OH-damaged and 10-HDA-treated VSMCs compared with that of the control using the 3-(4,5-dimethylthiazol-2-yl)-2,5-diphenyltetrazolium bromide (MTT) assay (*n* = 6, **P < 0.01, ***P < 0.001). **(C)** The malondialdehyde (MDA) levels in the media, indicating the degrees of VSMC lipid peroxidation (*n* = 5, *P < 0.05, ***P < 0.001). **(D)** The comparison of glutathione (reduced) content after VSMCs was treated by •OH or •OH + 10-HDA (*n* = 5, *P < 0.05, ***P < 0.001). The data are shown as mean ± standard deviation (SD).

To uncover whether 10-HDA rescues •OH-induced VSMC damage, the VSMC morphology was analyzed. Compared with the controls, the cellular shape of the VSMCs with •OH treatment was destroyed. With 10-HDA supplementation, the deformation of VSMCs was alleviated (Figure 3A). Furthermore, using an MTT assay, the significantly reduced VSMC viability by •OH (*P* < 0.001) was significantly recovered by 10-HDA (*P* < 0.01) (Figure 3B). In addition, the MDA level in the VSMC supernatant was significantly increased with an •OH challenge (*P* < 0.001), suggesting that lipid peroxidation of VSMCs occurred and was released from the cells; this effect was significantly reduced by 10-HDA (*P* < 0.05) (Figure 3C). Similarly, the MDA content within VSMCs was also significantly enhanced by •OH (*P* < 0.05) and significantly reduced with the addition of 10-HDA (*P* < 0.05) (Supplementary Figure S3). The results demonstrate that 10-HDA maintained VSMC viability against •OH toxicity, which may be the result of the inhibitory effect of 10-HDA on lipid peroxidation of VSMCs. Notably, the reduced glutathione in VSMCs was significantly lessened under the oxidative stress of •OH (*P* < 0.001), which was significantly recovered by 10-HDA (*P* < 0.05) (Figure 3D), indicating that 10-HDA may relieve oxidative stress in blood vessels.

### 10-HDA Restored the VSMC Proteome Altered by •OH Challenge

**Figure 4:**
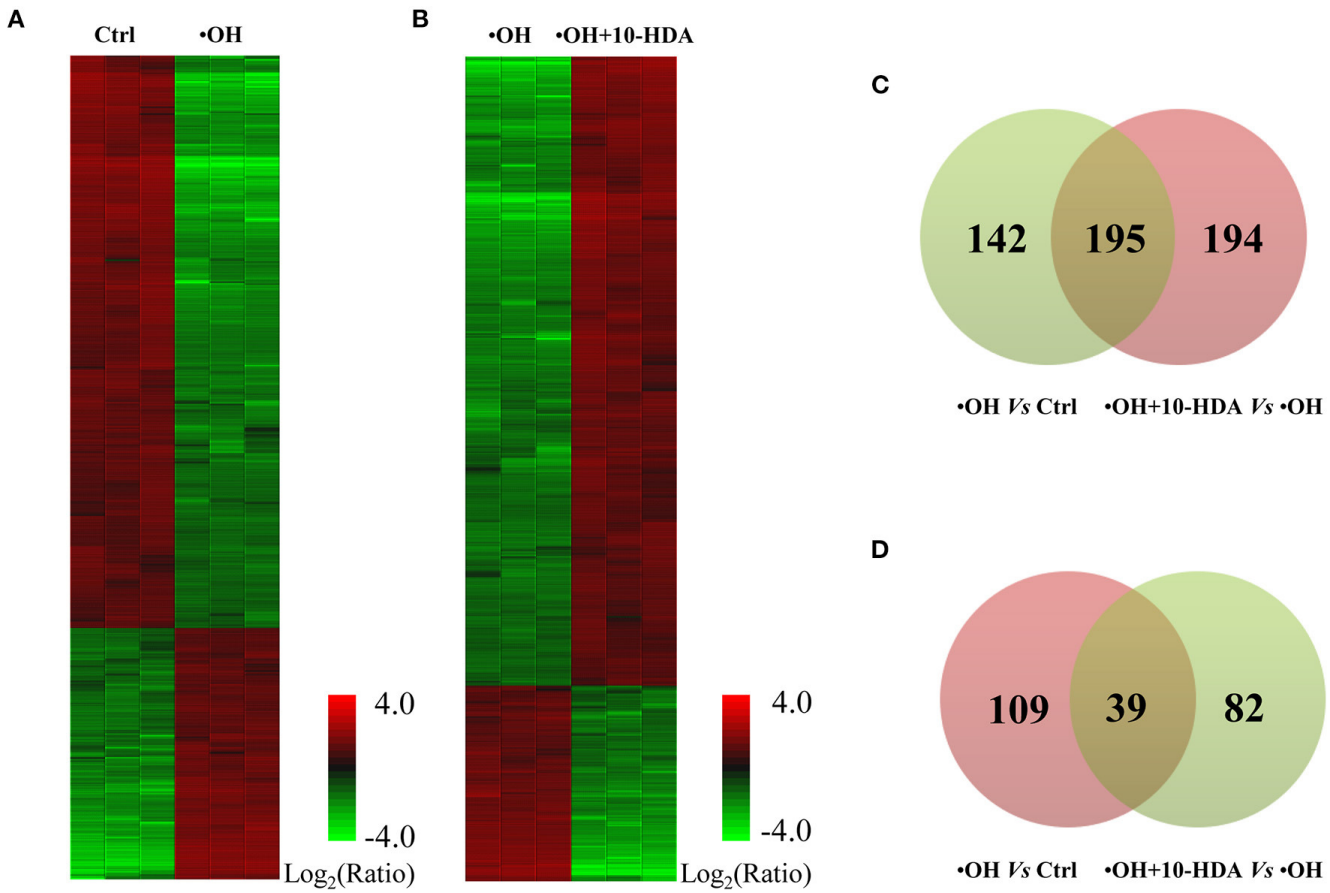
10-Hydroxydec-2-enoic acid (10-HDA) rescues the expression of a series of proteins affected by •OH in VSMCs. **(A)** The heatmap shows the significantly downregulated and upregulated proteins in the •OH-damaged VSMCs compared with those in the controls. **(B)** The differentially expressed proteins clustered between •OH-damaged and 10-HDA-supplemented VSMCs. **(C)** The Venn diagram indicating the proteins downregulated by •OH that were upregulated with 10-HDA treatment in VSMCs. **(D)** The number of proteins upregulated by •OH that can also be downregulated by 10-HDA. The number in the light green or pink circle indicates the proteins that are downregulated or upregulated, respectively.

To further explore the role of 10-HDA in protecting VSMC viability at the molecular level, the differentially expressed proteins regulated by •OH and 10-HDA were compared. In total, 3,401, 2,883, and 3,467 proteins were identified in the control, •OH-damaged, and 10-HDA-supplemented VSMCs, respectively. Compared with the proteins in controls, 337 proteins were significantly downregulated, and 148 proteins were significantly upregulated in the •OH-damaged VMSCs (Figure 4A and Supplementary Table S1). However, with the addition of 10-HDA to the •OH-damaged VMSCs, 121 proteins were significantly downregulated, and 389 proteins were significantly upregulated (Figure 4B and Supplementary Table S2). Notably, 195 proteins downregulated by •OH were upregulated with 10-HDA treatment (Figure 4C), whereas only 39 proteins upregulated by •OH were downregulated by 10-HDA treatment (Figure 4D). Therefore, 10-HDA may restore the expression of a wide spectrum of proteins that were suppressed by •OH.

### 10-HDA Reduced •OH-Induced Cytotoxicity by Elevating Protein and Energy Metabolism

**Figure 5:**
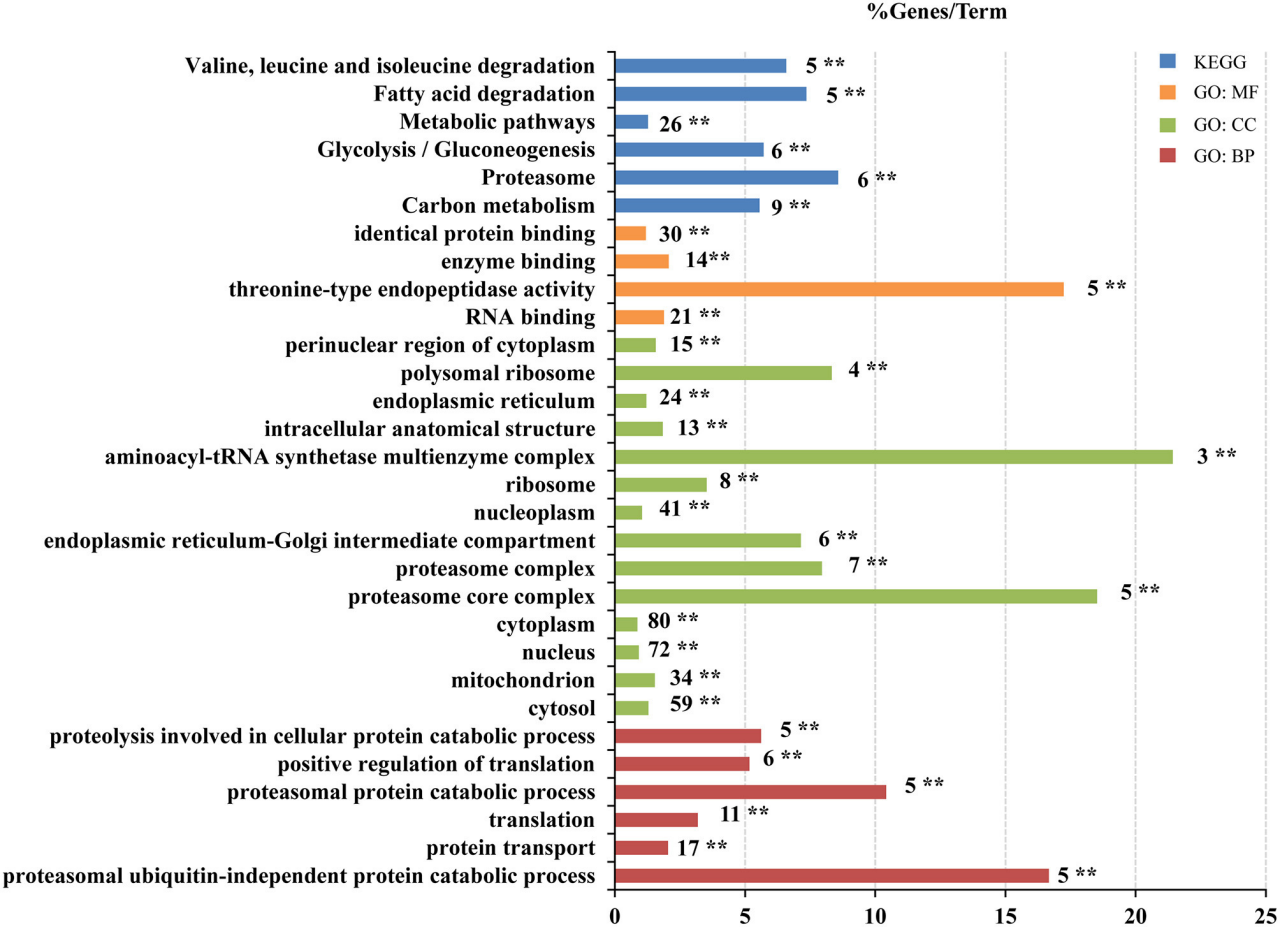
10-Hydroxydec-2-enoic acid (10-HDA) reinforces the cascades that are disrupted by •OH in VSMCs. Pathway enrichment analysis of the 195 proteins downregulated by •OH but upregulated by 10-HDA using KOBAS-i. The Gene Ontology (GO) term or Kyoto Encyclopedia of Genes and Genomes (KEGG) pathway is marked near the bar, and **indicates the corrected *P-*value <0.01 that represents the significance of the term or pathway. The bar length indicates the % Genes/Term, which is the percentage of the input proteins to the background.

To reveal the biological functions of the VSMC differentially expressed proteins influenced by •OH and 10-HDA, 195 proteins were analyzed for GO term and KEGG pathway enrichment. As a result, 54 items were significantly enriched across the following categories, namely, 14 in GO biological process (BP), 21 in GO cellular component (CC), 10 in GO molecular function (MF), and 9 in KEGG pathways (Supplementary Table S3). The GO terms and KEGG pathways with *P* < 0.01 are primarily involved in protein and energy metabolism, such as translation, protein transport, ribosome, RNA binding, proteasomal protein catabolic processes, fatty acid degradation, and glycolysis/gluconeogenesis (Figure 5). Hence, 10-HDA may rescue protein and energy metabolism pathways to safeguard VSMC viability when resisting •OH toxicity. The capability of 10-HDA to reverse energy production was evidenced by the significantly decreased ATP levels in VSMCs under •OH stress (*P* < 0.01), which was significantly increased with supplemental 10-HDA (*P* < 0.05) (Supplementary Figure S4). However, the 39 proteins upregulated by •OH and downregulated by 10-HDA were only enriched in four GO terms (NADP metabolic process, RNA binding, poly(A) + mRNA export from the nucleus, and nuclear inclusion body), and none of the KEGG pathways (Supplementary Figure S5).

### 10-HDA-Rescued VSMC Proteins Were Mainly Located in the Nucleus, Cytoplasm, and Mitochondrion

**Figure 6:**
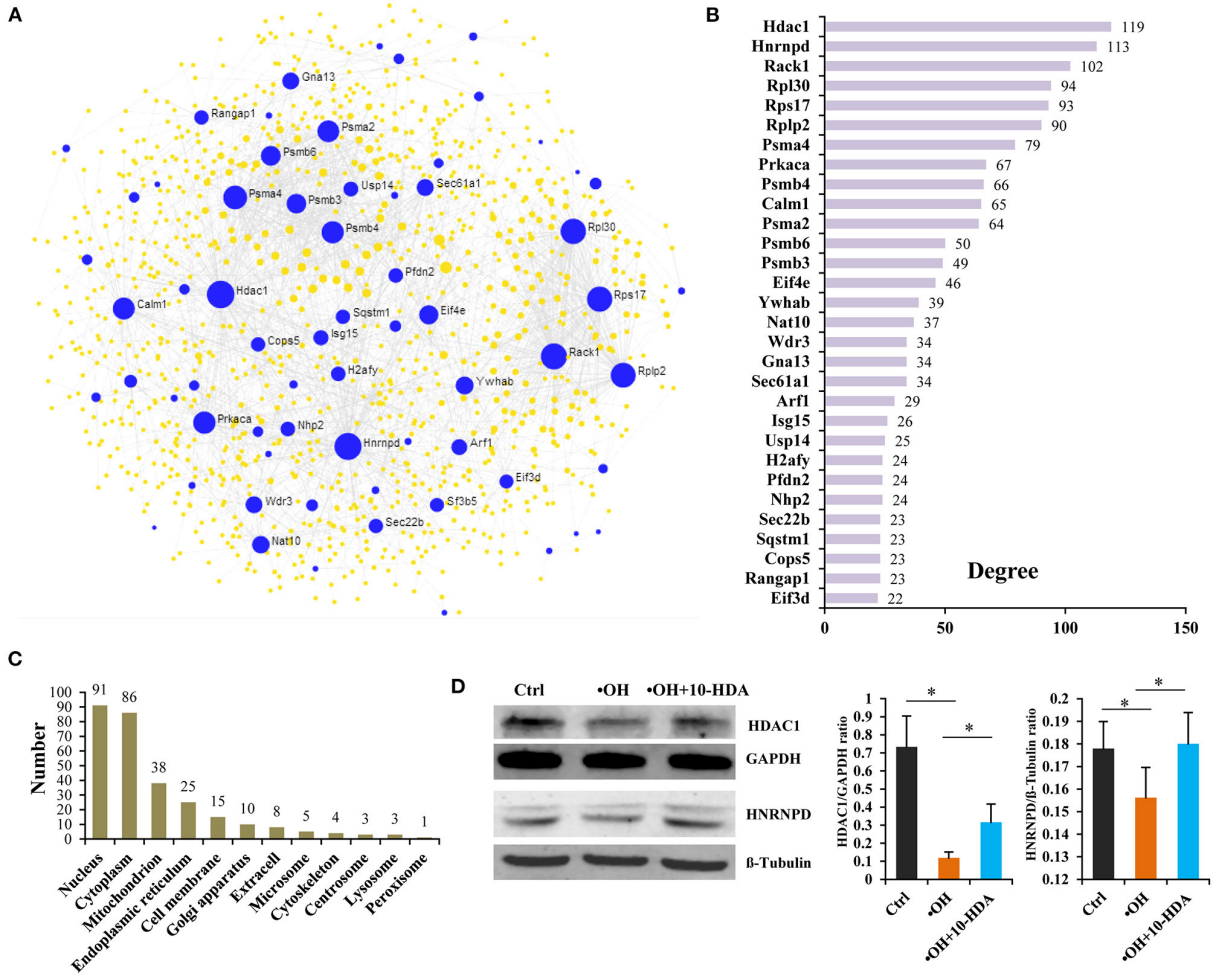
10-Hydroxydec-2-enoic acid (10-HDA) restores the expression of a set of proteins downregulated by •OH that are functionally connected and broadly distributed in VSMCs. **(A)** The “continent” (Subnetwork 1) of the protein–protein interaction (PPI) network constructed by NetworkAnalyst 3.0 using the 195 proteins downregulated by •OH and upregulated with 10-HDA supplementation, in which the blue dots represent the seeds (the proteins downregulated by •OH and upregulated by 10-HDA treatment in VSMCs) among the nodes. The area of the dot symbolizes the degree of connectivity of the protein node in the PPI network. **(B)** The top 30 hub nodes with high degrees in subnetwork 1. **(C)** The numbers of the predicted subcellular localizations by Cell-PLoc 2.0 for the VSMC proteins downregulated by •OH and upregulated by 10-HDA. **(D)** The comparisons (shown as mean ± SD) of histone deacetylase 1 (HDAC1) and heterogeneous nuclear ribonucleoprotein D0 (HNRNPD) expressions in control, •OH-damaged, and 10-HDA-supplemented VSMCs through Western blotting analysis (*n* = 3, *P < 0.05).

To establish the functional connections between VSMC proteins that were impacted by •OH but recovered by 10-HDA, and to identify key node proteins, a PPI network was constructed. The 195 proteins generated 18 subnetworks, including a “continent” (i.e., subnetwork 1) (Figure 6A) and 17 “islands” (i.e., subnetworks 2–18) (Supplementary Figure S6). Subnetwork 1 had 66 seeds, 1,090 nodes, and 1,790 edges. The top 30 hub nodes (also the seeds) with their degrees are shown in Figure 6B. These nodes with high connectivity in the PPI network may be the key proteins influenced by •OH and 10-HDA. Protein subcellular localization predicted that the proteins were distributed across the cell from the cell membrane to the nucleus, particularly in the nucleus, cytoplasm, and mitochondrion (Figure 6C and Supplementary Table S4), the major locations for protein synthesis and transport and energy metabolism. Furthermore, Western blotting confirmed that HDAC1 and HNRNPD (the top two hub nodes in the PPI networks) were significantly downregulated by •OH and significantly upregulated *via* the supplementation of 10-HDA (*P* < 0.05) (Figure 6D), as in the proteomic analysis.

## Discussion

10-Hydroxydec-2-enoic acid, a unique ingredient in RJ beneficial to health, has been characterized by its ability to prevent cell damage (7–9). To unveil the mechanisms of 10-HDA's protective qualities, its role in eliminating ROS was investigated. 10-HDA demonstrated the ability to scavenge •OH, the most harmful ROS, *via* direct oxidation reactions with •OH. In •OH-damaged mouse VSMCs, 10-HDA restored cell viability by reducing lipid peroxidation, increasing glutathione levels, and rescuing protein and energy metabolism in the nucleus, cytoplasm, and mitochondrion. Our results shed light on the potential of 10-HDA in the prevention and treatment of vascular diseases related to •OH-induced VSMC damage.

10-Hydroxydec-2-enoic acid has direct reactions with •OH, which was confirmed by detecting the products after the addition of 10-HDA to the Fenton reagent. With both an –OH group and a C=C bond, 10-HDA can be easily oxidized. Specifically, –OH can be oxidized to C=O or –COOH, and hydroxylation of the C=C bond can be catalyzed through oxidation (28, 29). According to the UHPLC-HRMS data, the molecular weights of a series of chemical compounds detected were in accordance with the molecular weights of the predicted oxidation products, indicating that 10-HDA may be oxidized to different products *via* multiple reaction steps with •OH. Hence, in the presence of •OH, 10-HDA can play the role of a “sponge” that absorbs oxygen atoms and therefore eliminates •OH.

Since 10-HDA likely scavenges •OH, this could be the molecular mechanism of 10-HDA's health-promoting functions. A surplus of ROS is the fundamental cause of many types of cardiovascular diseases. For example, atherosclerosis can occur when the viability of VSMCs is impaired, as damage to VSMCs can lead to vascular calcification (30). Atherosclerosis is also considered a chronic inflammatory disease, in which oxidative stress due to ROS is the critical underlying factor (31). High blood pressure is closely related to abnormal VSMC contraction and relaxation, and it has been documented that oxidative stress is a common mediator underlying the pathophysiological processes of hypertension (32). Therefore, the fact that reduced VSMC viability by •OH was reversed by 10-HDA implies that 10-HDA holds the potential to protect the cardiovascular system *via* the removal of •OH. Hence, the intake of 10-HDA may mitigate the symptoms of atherosclerosis and hypertension. Moreover, ROS accumulation stimulated by LPS in murine macrophages is often applied as an inflammatory model (4), and LPS-induced mitochondrial ROS in cardiomyocytes is correlated with myocarditis (33, 34). Given that 10-HDA has been reported to alleviate the LPS-induced inflammation *in vitro* and *in vivo* (4, 5), our evidence of 10-HDA removing ROS suggests a role in inhibiting inflammation and inflammation-related diseases. In addition, we confirmed that 10-HDA is capable of protecting VSMC viability by inhibiting lipid peroxidation of VSMCs. Collectively, the above evidence concludes that 10-HDA is a potential agent for remedying ROS-derived human illnesses.

Our proteomic results further revealed the underlying mechanism of 10-HDA against •OH toxicity in VSMCs. First, the abnormal expression and degradation of cellular proteins driven by •OH were remedied by 10-HDA. In this study, within the GO terms and KEGG pathways enriched by the 195 proteins that were upregulated by 10-HDA against •OH, a cascade of processes involved in protein metabolism were included, such as translation, protein transport, ribosome, and RNA binding, which are essential for cellular protein production. This suggests that protein synthesis disrupted by ROS could be reversed by 10-HDA (35). ROS impairs proteasome function, which is essential in the removal of oxidized proteins to maintain normal cellular behavior (36); proteasome dysfunction results in proteome instability (37). Therefore, the •OH-induced proteasomal suppression could impair cellular viability. In this study, multiple pathways of protein degradation were enriched, including the following GO terms: proteasomal protein catabolic process, proteasomal ubiquitin-independent protein catabolic process, proteasome complex, and proteasome, indicating that the impairment caused by •OH on regular protein degradation in VSMCs could be mitigated by 10-HDA. In addition, the proteins downregulated by 10-HDA in the •OH-damaged VSMCs were enriched in the GO term poly(A) + mRNA export from the nucleus, a process that promotes nuclear RNA decay (38). Therefore, 10-HDA might prevent •OH-induced RNA destruction in VSMCs. All this evidence suggests that 10-HDA can reduce the adverse effect of •OH *via* multiple pathways.

Second, the energy production inhibited by •OH was restored by 10-HDA. Carbohydrates are fundamental for generating ATP for cell survival and growth (39). In this study, the enriched glycolysis/gluconeogenesis pathway indicates that the treatment of •OH may disrupt the glycolytic process in VSMCs. Fatty acids are another important source of energy *via* multiple steps of metabolism (40). The significantly enriched fatty acid degradation pathway in the current data also suggests the impairment of the energy supply by oxidative damage. Our results established that the decreased intracellular ATP levels of VSMCs by •OH due to these impairments can be reversed by 10-HDA supplementation. Notably, the concept that aerobic glycolysis and fatty acid oxidation metabolic pathways in VSMCs are crucial to functional vasculature is broadly accepted (41); thus, 10-HDA is a promising treatment for VSMC-related vascular diseases.

Third, a large number of the 10-HDA upregulated proteins mainly function in the cell nucleus, cytoplasm, mitochondrion, and endoplasmic reticulum. 10-HDA-rescued proteins were found widely distributed within VSMCs from the cell membrane, throughout the cytoplasm, and in the nucleus, indicating that 10-HDA could provide broad protection against •OH damage at the molecular level. However, most of these proteins were located in the nucleus and cytoplasm, which is logical because the key impact of ROS is nuclear DNA fragmentation and chromatin dysfunction (42). Therefore, one of the major effects of 10-HDA in VSMCs is to protect the nuclear and cytoplasmic proteins, which is also reflected by the PPI results in which more than 90% of the top 30 hub nodes were predicted to locate in the nucleus and cytoplasm. Moreover, the enrichment in the mitochondrion and endoplasmic reticulum further supports the fact that protecting protein and energy metabolism are major mechanisms through which 10-HDA reduces •OH toxicity. In addition, our PPI results also provide vital target proteins involved in 10-HDA's amelioration of vascular abnormality. Of the top two hub nodes with degree >110, HDAC1 can increase VSMC viability and migration under quiescent conditions (43). Although the knowledge of the role of HNRNPD in VSMCs is very limited, HNRNP family members are shown to be important modulators of VSMC functions (44, 45). Therefore, HDAC1 and HNRNP may be the crucial intracellular molecules associated with 10-HDA's restoration of VSMC survival.

## Conclusion

In conclusion, this work provides concrete evidence that 10-HDA, a unique RJ component, has a biological function in scavenging •OH *via* oxidative reactions. Using •OH-damaged mouse VSMCs as a model, we confirmed the function of 10-HDA in protecting VSMC viability against •OH-induced cytotoxicity. Specifically, the cytoprotective mechanism of 10-HDA against •OH is believed to restore protein and energy metabolism within the nucleus, cytoplasm, and mitochondrion. Our results shed light on the novel potential of 10-HDA in the prevention and treatment of cardiovascular diseases in human.

## Data Availability Statement

The datasets presented in this study can be found in online repositories. The names of the repository/repositories and accession number(s) can be found in the article/Supplementary Material. The data sets (MS proteomic row data) for this study can be found in the ProteomeXchange Consortium (http://proteomecentral.proteomexchange.org) with the dataset identifier IPX0003749001.

## Author Contributions

PF: conceptualization, methodology, investigation, and writing—original draft. FS: investigation. CM: methodology and investigation. QW: investigation. YZ: investigation. JS: investigation. JF: investigation. LZ: investigation, funding acquisition, and supervision. BH: methodology, investigation, funding acquisition, and writing—review and editing. JL: project administration, funding acquisition, writing—review and editing, and supervision. All authors contributed to the article and approved the submitted version.

## Funding

This study was supported by the open project fund from the Key Laboratory of Pollinating Insect Biology, Ministry of Agriculture and Rural Affairs, P. R. China (2018MFNZS02), the National Natural Science Foundation of China (32070742 and 31970428), the Innovative Funds Plan of Henan University of Technology (2021ZKCJ16), and the Modern Agro-Industry Technology Research System (CARS-45) in China.

## Conflict of Interest

The authors declare that the research was conducted in the absence of any commercial or financial relationships that could be construed as a potential conflict of interest.

## Publisher's Note

All claims expressed in this article are solely those of the authors and do not necessarily represent those of their affiliated organizations, or those of the publisher, the editors and the reviewers. Any product that may be evaluated in this article, or claim that may be made by its manufacturer, is not guaranteed or endorsed by the publisher.

## Supplementary Material

Supplementary Figure S1 10-HDA scavenges •OH *via* Fenton/Griess reagent colorimetric assay. Unlike the Fenton/1,10-phenanthroline monohydrate-based method, it requires the ethanol-free samples according to the kit's instruction. Here, 10-HDA was dissolved in olive oil with the content of 2% (g/mL), and the same volume of olive oil without 10-HDA was the control (*n* = 3, ^***^*P* < 0.001).

Supplementary Figure S2 The MS base peaks implicated in the extra chromatographic peaks emerged by the treatment of •OH to 10-HDA. **(A)** shows the MS base peak for 10-HDA without or with •OH addition. **(B)** displays the emerged MS base peaks after the reaction of •OH and 10-HDA.

Supplementary Figure S3 10-HDA reduces the intracellular MDA content of VSMCs, an indicator for lipid peroxidation, that are enhanced by •OH. The data were shown as mean ± SD (*n* = 4, ^*^*P* < 0.05).

Supplementary Figure S4 10-HDA boosts the intracellular ATP level in VSMCs that are inhibited by the oxidative stress from •OH (data shown as mean ± SD with *n* = 3, ^**^*P* < 0.01, ^*^*P* < 0.05).

Supplementary Figure S5 10-HDA may suppress the functions of NADP metabolic process, poly(A) + mRNA export from nucleus, RNA binding, and nuclear inclusion body induced by •OH in VSMCs. The items shown are the GO terms enriched by the 39 proteins that are up-regulated by •OH and down-regulated by 10-HDA. The number of the proteins implicated in the items is near each bar, and ^*^ means the corrected *P*-value <0.05. % Genes/Term, the bar length, is the ratio of input proteins to the all within a term.

Supplementary Figure S6 The Subnetworks 2–18 of the PPI network, known as the “islands”, constructed by the VSMC proteins down-regulated by •OH and up-regulated by 10-HDA. The seeds of the networks were shown as the blue dots among nodes. The area of the node represents its degree in the network.

Supplementary Table S1 The differentially expressed proteins by •OH in VSMCs.

Supplementary Table S2 The differentially expressed proteins with the addition of 10-HDA to the •OH treated VSMCs.

Supplementary Table S3 GO term and KEGG pathway enrichment for the proteins down-regulated by •OH and up-regulated with 10-HDA treatment in VSMCs.

Supplementary Table S4 The subcellular localization prediction for the proteins down-regulated by •OH and up-regulated by 10-HDA administration in VSMCs.
