# Supplementary figures and images for: 10-Hydroxydec-2-Enoic Acid Reduces Hydroxyl Free Radical-Induced Damage to Vascular Smooth Muscle Cells by Rescuing Protein and Energy Metabolism
Source: Front Nutr. 2022 May 26;9:873892. doi: 10.3389/fnut.2022.873892 (PMC9196250; doi:10.3389/fnut.2022.873892)

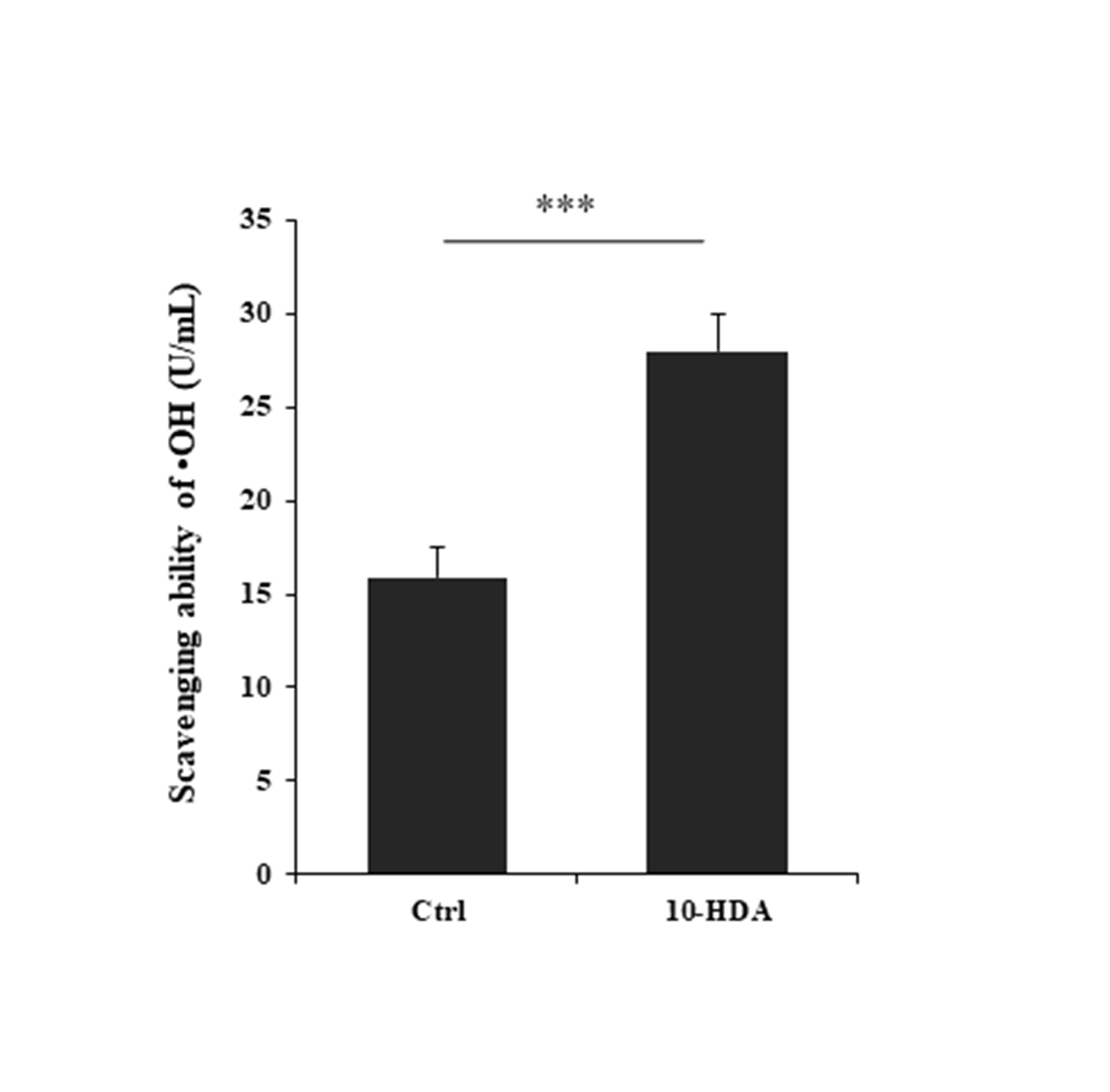

Supplement: Supplementary Figure S1 — 10-HDA scavenges •OH via Fenton/Griess reagent colorimetric assay. Unlike the Fenton/1,10-phenanthroline monohydrate-based method, it requires the ethanol-free samples according to the kit's instruction. Here, 10-HDA was dissolved in olive oil with the content of 2% (g/mL), and the same volume of olive oil without 10-HDA was the control (n = 3, ***P < 0.001). [file Image_1.TIF]

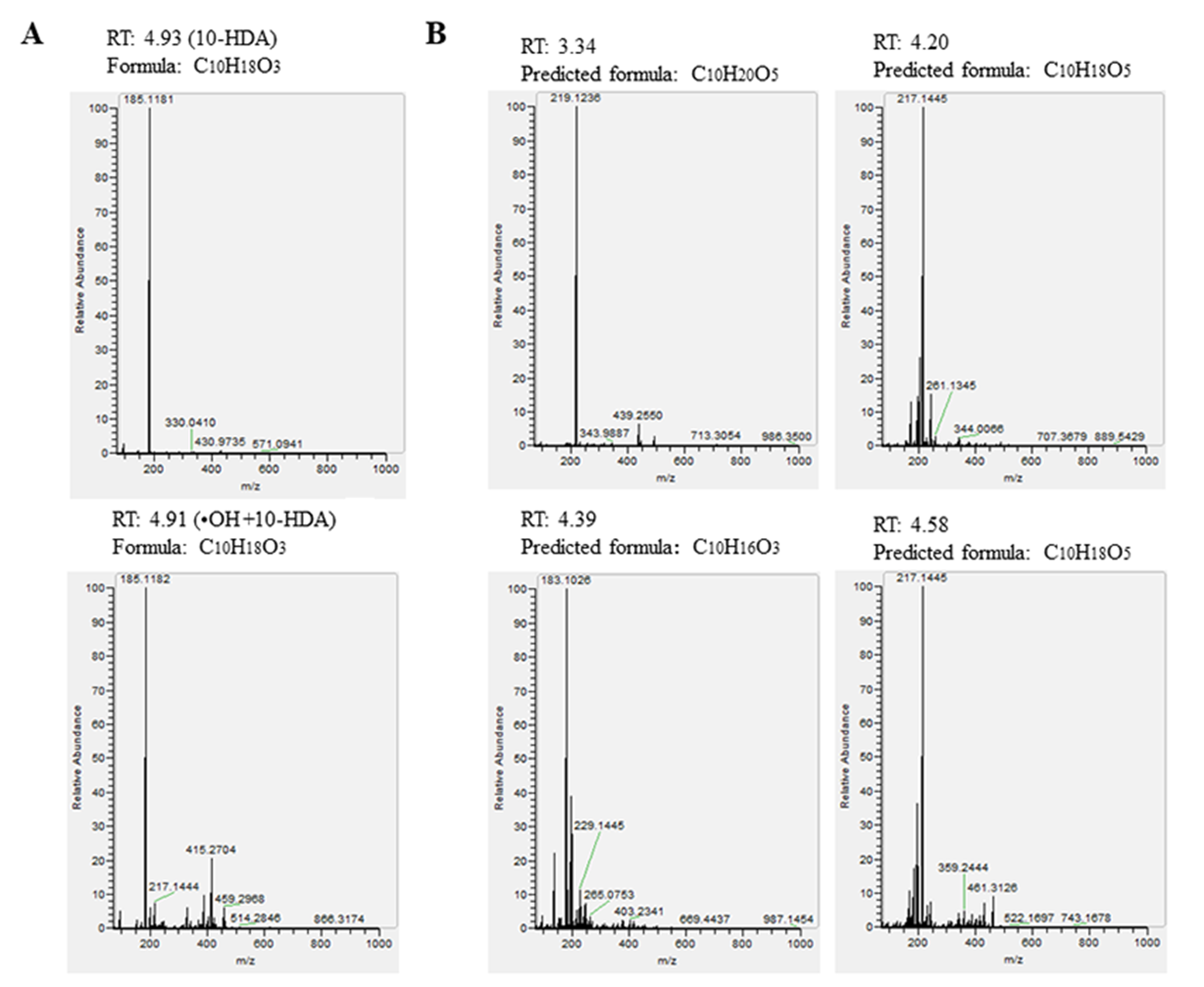

Supplement: Supplementary Figure S2 — The MS base peaks implicated in the extra chromatographic peaks emerged by the treatment of •OH to 10-HDA. (A) shows the MS base peak for 10-HDA without or with •OH addition. (B) displays the emerged MS base peaks after the reaction of •OH and 10-HDA. [file Image_2.TIF]

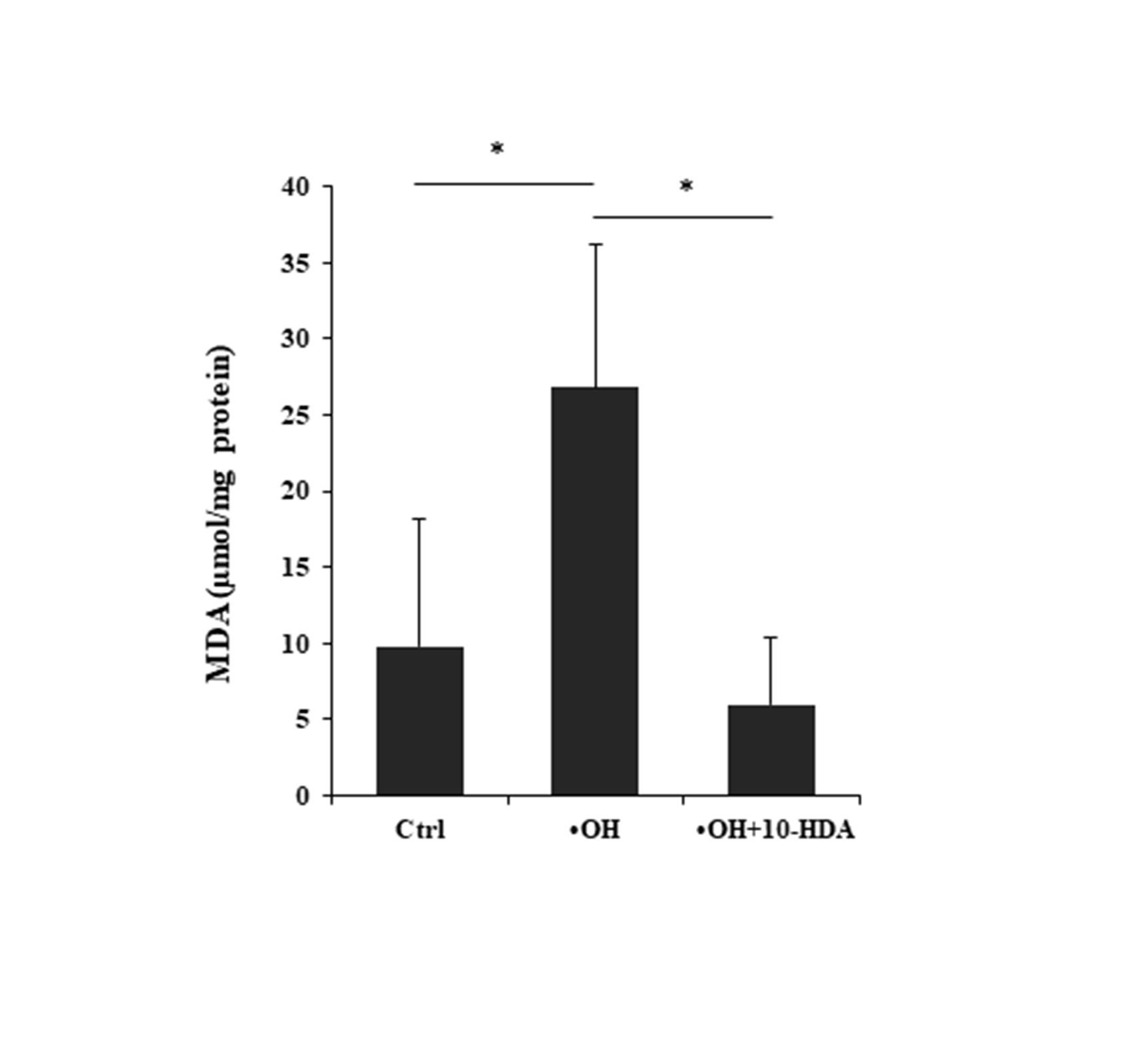

Supplement: Supplementary Figure S3 — 10-HDA reduces the intracellular MDA content of VSMCs, an indicator for lipid peroxidation, that are enhanced by •OH. The data were shown as mean ± SD (n = 4, *P < 0.05). [file Image_3.TIF]

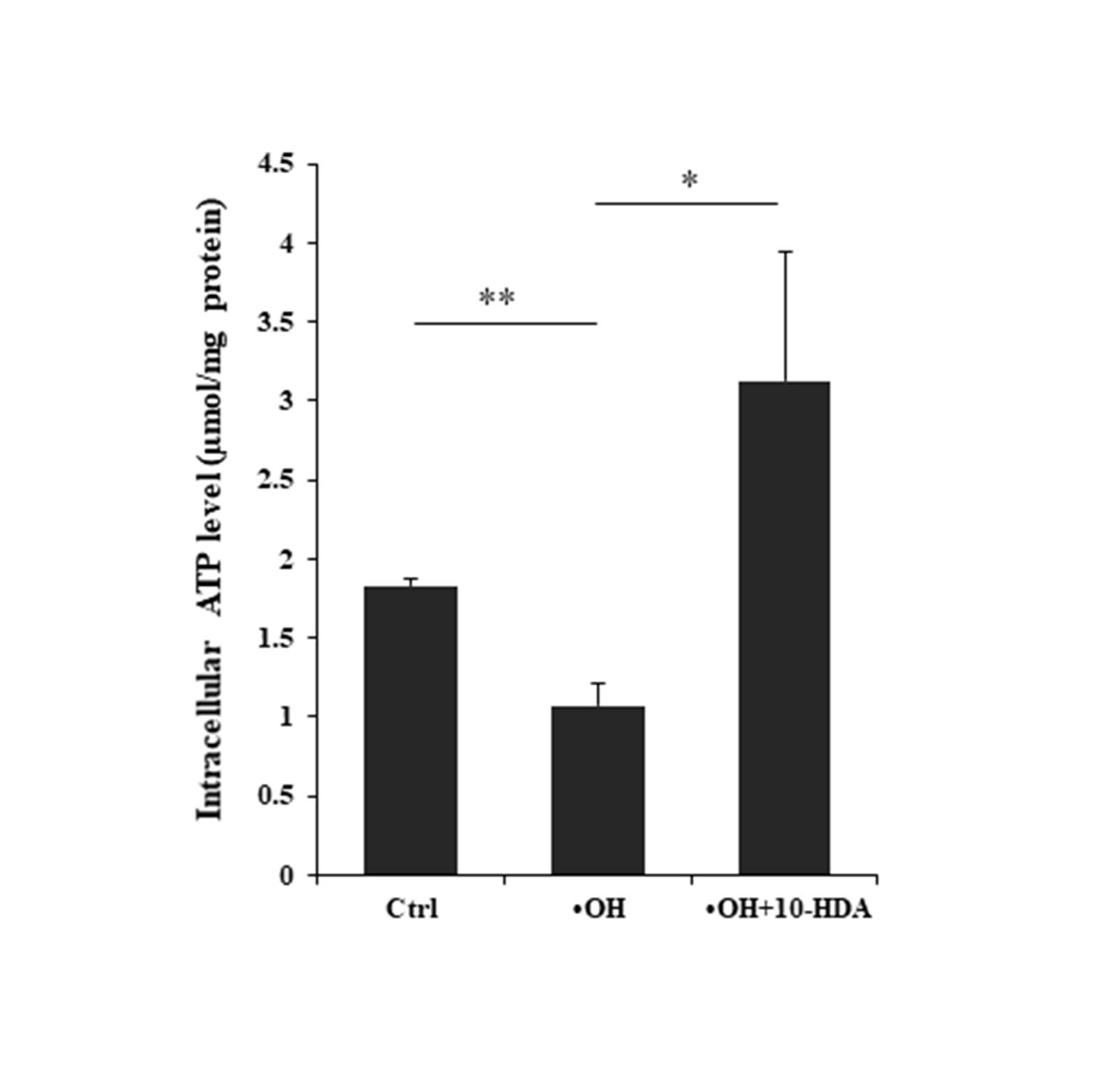

Supplement: Supplementary Figure S4 — 10-HDA boosts the intracellular ATP level in VSMCs that are inhibited by the oxidative stress from •OH (data shown as mean ± SD with n = 3, **P < 0.01, *P < 0.05). [file Image_4.TIF]

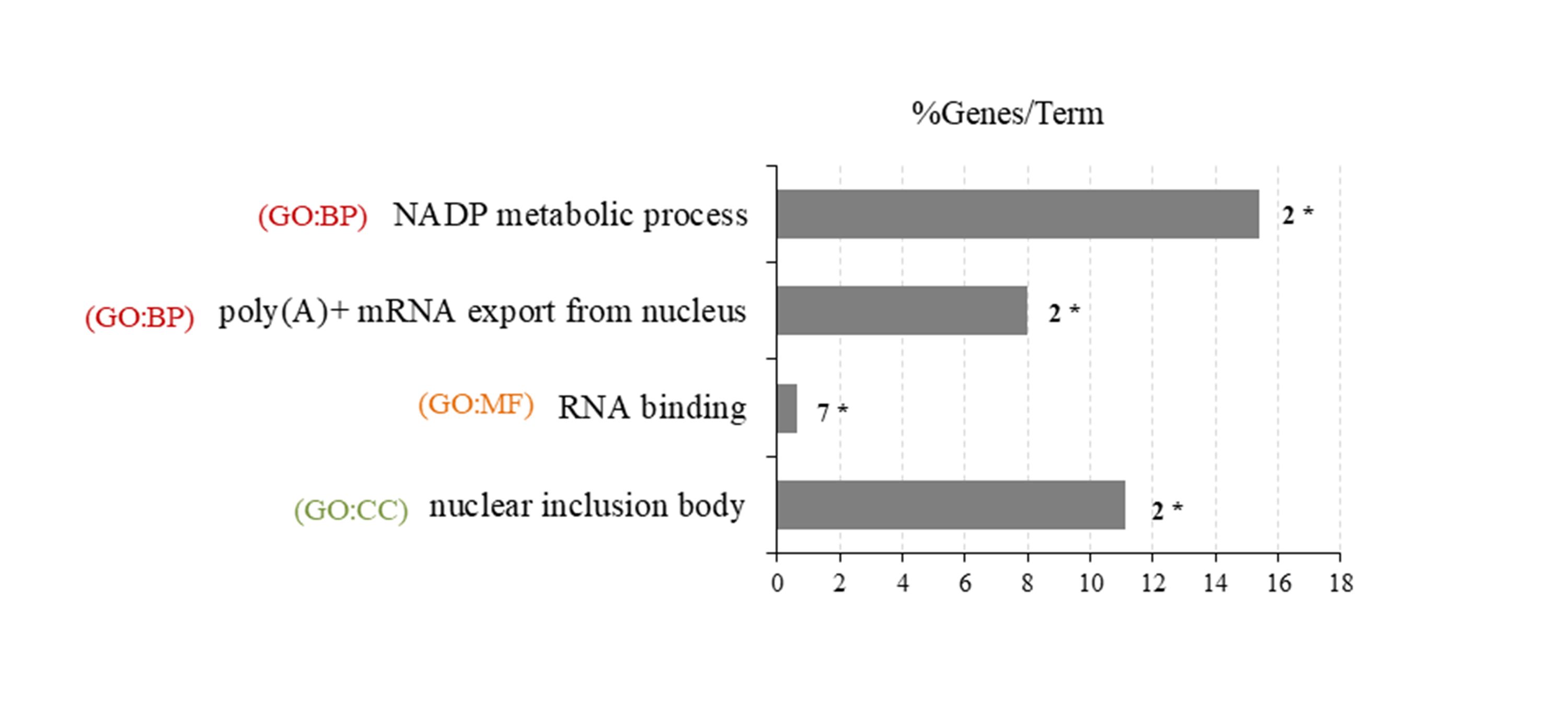

Supplement: Supplementary Figure S5 — 10-HDA may suppress the functions of NADP metabolic process, poly(A) + mRNA export from nucleus, RNA binding, and nuclear inclusion body induced by •OH in VSMCs. The items shown are the GO terms enriched by the 39 proteins that are up-regulated by •OH and down-regulated by 10-HDA. The number of the proteins implicated in the items is near each bar, and * means the corrected P-value <0.05. % Genes/Term, the bar length, is the ratio of input proteins to the all within a term. [file Image_5.TIF]

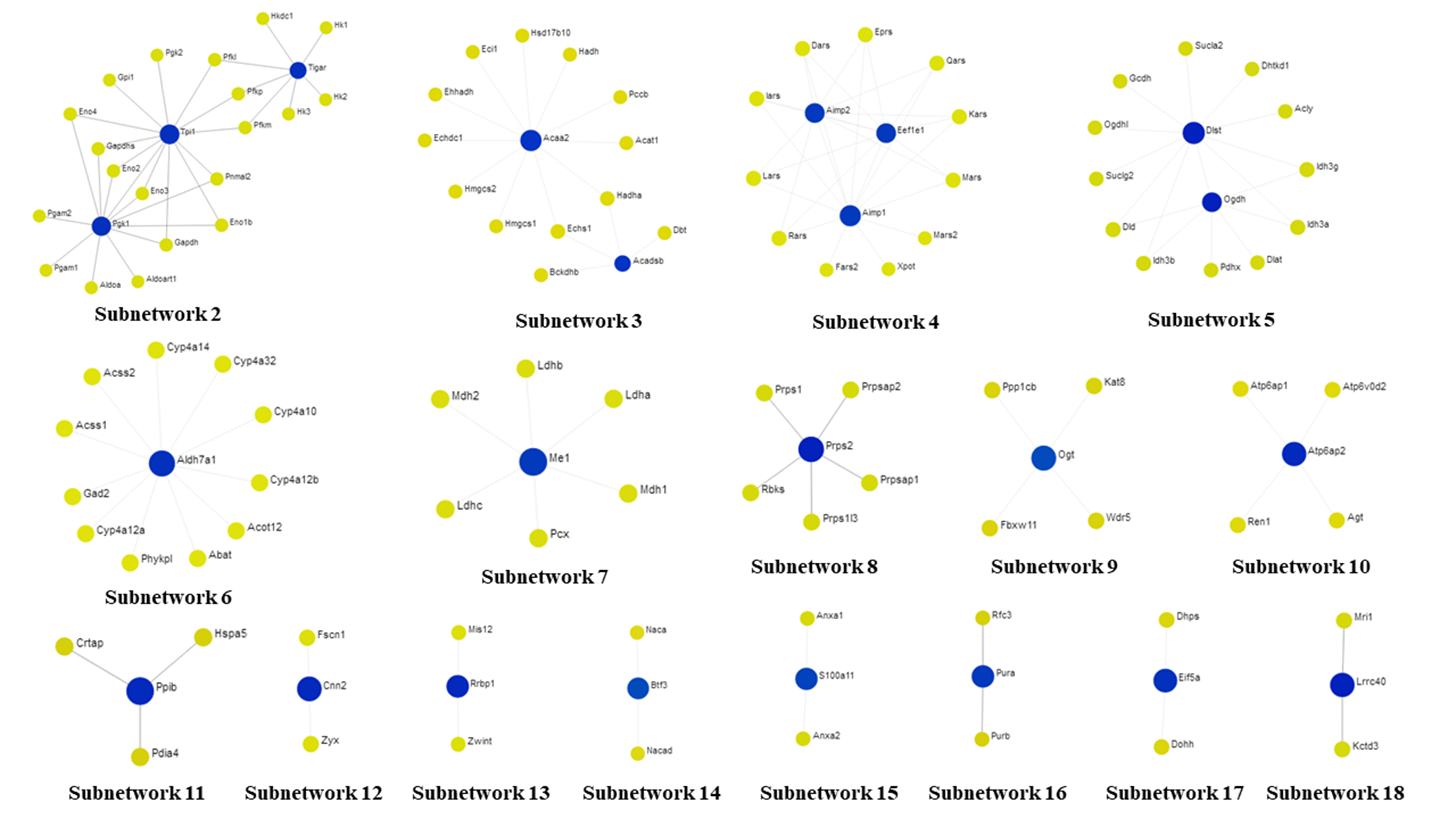

Supplement: Supplementary Figure S6 — The Subnetworks 2–18 of the PPI network, known as the “islands”, constructed by the VSMC proteins down-regulated by •OH and up-regulated by 10-HDA. The seeds of the networks were shown as the blue dots among nodes. The area of the node represents its degree in the network. [file Image_6.TIF]
